# Synthesis and Anticancer Activity of Some New *S*-Glycosyl and *S*-Alkyl 1,2,4-Triazinone Derivatives

**DOI:** 10.3390/molecules16075682

**Published:** 2011-07-04

**Authors:** Hosam A. Saad, Ahmed H. Moustafa

**Affiliations:** 1 Department of Chemistry, Faculty of Science, Taif University, Taif, 21974, Kingdom of Saudi Arabia; 2 Department of Chemistry, Faculty of Science, Zagazig University, Zagazig, 44511, Egypt

**Keywords:** *S*-glycosyl, *S*-alkyl triazine, 1,2,4-triazinone, thiadiazolotriazines, anticancer activities

## Abstract

A series of S-glycosyl and S-alkyl derivatives of 4-amino-3-mercapto-6-(2-(2-thienyl)vinyl)-1,2,4-triazin-5(4H)-one (**1**)were synthesized using different halo compounds such as preacetylated sugar bromide, 4-bromobutylacetate, 2-acetoxyethoxy-methyl bromide, 3-chloropropanol, 1,3-dichloro-2-propanol, epichlorohydrin, allyl bromide, propargyl bromide, phthalic and succinic acids in POCl_3_. The structures of the synthesized compounds have been deduced from their elemental analysis and spectral (IR, ^1^H-NMR, and^ 13^C-NMR) data. Some of the synthesized compounds were screened as anticancer agents. Significant anticancer activities were observed *in vitro* for some members of the series, and compounds 4-Amino-3-(3-hydroxypropylthio)-6-(2-(2-thienyl)vinyl)-1,2,4-triazin-5(4H)-one (**12**) and 3-(4-Oxo-3-(2-(2-thienyl)vinyl)-4H-[1,3,4]thiadiazolo-[2,3-c][1,2,4]tr-iazin-7-yl)propanoic acid (**18**) are active cytotoxic agents against different cancer cell lines.

## 1. Introduction

The chemistry and diverse applications of heterocyclic glycosyl derivatives have received much attention due to their pronounced biological activity. 6-Aza analogues of the naturally occurring nucleic acids components represent isosteres with the C-6 carbon atom of the pyrimidine nucleoside base being replaced by nitrogen [1]. They became attractive to scientists after it was discovered that 6-azauracil and 6-azauridine are potent inhibitors of tumor growth, and it was determined that 6-azauridine is converted in the organism into 6-azauridylphosphate which blocks the enzyme orotic acid decarboxylase, and thus the conversion of orotidylic acid into uridylic acid is impaired [2,3]. 2-Glycosyl derivatives of 1,2,4-triazine-3,5(2*H*,4*H*)-diones and their thiones (6-azauracil derivatives) possess [4,5,6] biological activity as cytotoxic, antiviral, enzyme inhibiting, immunosuppressive, antiphlogestic and bacteriostatic agents [7,8]. As will as, thiophene-containing compounds are well known to exhibit various biological activities such as anti-HIV PR inhibitors [9], anti breast cancer (MCF-7) [10], anti-inflammatory [11,12,13]. anti-protozoal [14], antitumor [15], antitubercular with antimycobacterial activity [16]. On the other hand, the 1,2,4-triazine moiety has also attracted the attention of chemists because many 1,2,4-triazines are biologically active [17,18,19,20,21,22] and are used in medicine, especially as anti AIDS, anticancer [23,24], and antitubercular agents [25], for their anti-anxiety and anti-inflammatory activities [26,27], as well as in agriculture [28,29,30,31]. 

## 2. Results and Discussion

**Scheme 1:**
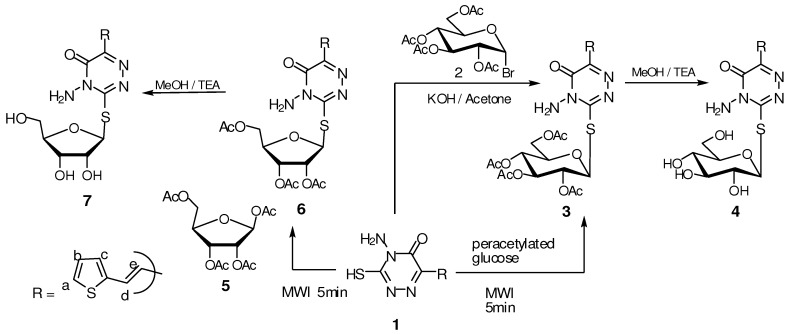
Synthesis of some *S*-glycosyl 1,2,4-triazinone.

For the above reasons we sought to synthesize a series of *S*-glycosyl and S-alkyl of 4-amino-3-mercapto-6-(2-(2-thienyl)vinyl)-1,2,4-triazin-5(4*H*)-one (**1**) [32] which should combine all the above benefits in one target and to then test the anticancer activity of these synthesized compounds against a panel of human cell lines including hepatocellular carcinoma (Hep-G2), colon carcinoma (HCT-116), and histiocytic lymphoma and breast adenocarcinoma (MCF-7) (ATCC, VA, USA). In our previous work we prepared compound **1** by two different methods [33]. The synthetic routes to *S*-glycosides **3–7** via microwave irradiation (MWI, 2,450 MHz, 800 W) and conventional methods are illustrated in Scheme 1. 

The appearance of signals at δ 1.87, 1.92, 1.95, 2.02 ppm due to the four CH_3_ of four OAc groups and at δ 6.21 ppm due to the anomeric proton of sugar with *J* = 9.60 Hz in the ^1^H-NMR confirms the formation of the *β*-configuration of *S*-glucoside **3**. This was also confirmed by the IR spectrum which showed a bands at 3,310, 1,739 and 1,660 cm^−1^ for NH_2_ and two ester and amide C=O groups.

The ^13^C-NMR spectrum of **3** showed signals at δ 61.63, 66.24, 68.47, 72.88, 73.13 ppm characteristic for sp^3^ carbon in a sugar moiety, in addition to δ 86.73 ppm for the anomeric carbon (C_1_). In compound **6 **the IR spectrum showed a band at 1,737 cm^−1^ for the acetoxy groups of the sugar and the ^1^H-NMR displayed a signal at δ 6.46 ppm with *J* = 2.80 Hz characteristic for the anomeric protons, in addition, inthe ^13^C-NMR spectrum the anomeric carbon appear at δ 87.6 ppm.

**Scheme 2:**
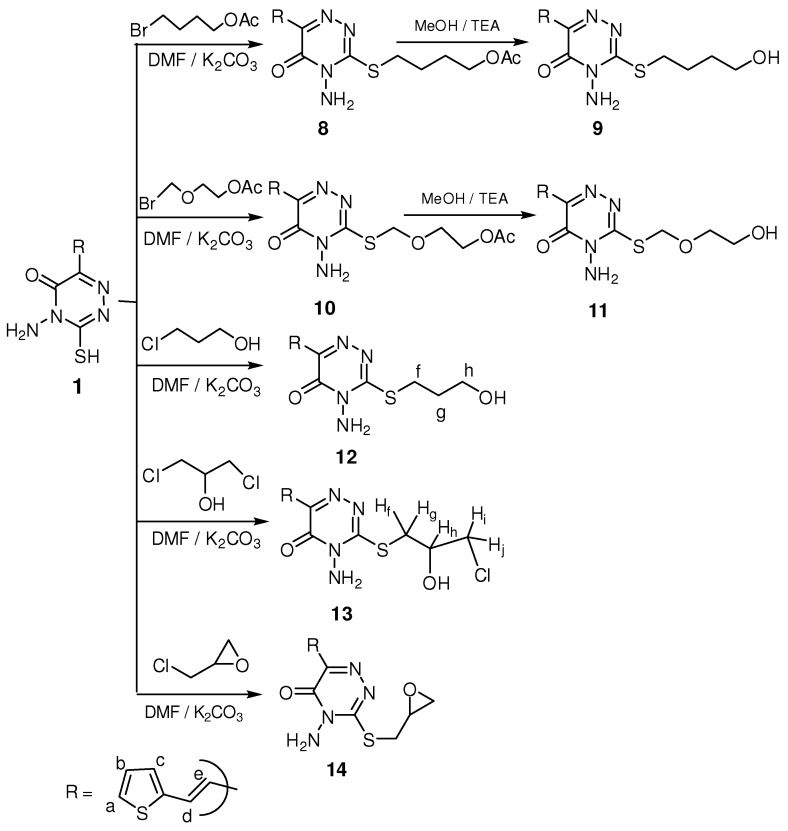
Synthesis of some *S* alkyl 1,2,4-triazinones.

The deacetylated compounds **4** and **7** showed in the IR spectra the disappearance of the acetoxy groups and the appearance of bands at 3,434 and 3,421 cm^−1^ for the resulting free hydroxyl groups. The ^1^H-NMR spectra and elemental analysis confirmed the structure as shown in the Experimental section. When compound **1** was treated with alkylating agents such as 4-bromobutylacetate, 2-acetoxy-ethoxymethyl bromide [34], 3-chloropropanol, 1,3-dichloro-2-propanol and epichlorohydrin using DMF as a solvent in the presence of K_2_CO_3_, acetyloxy *S*-alkyl 1,2,4-triazinones **8**, **10** and *S*-alkyl 1,2,4-triazinones **12-14** were obtained (Scheme 2). Deacetylation of compounds **8** and **10** in the presence of TEA/MeOH and few drops of water [35,36,37] yielded the deacetylated *S*-alkyl 1,2,4-triazinone **9 **and **11**, respectively (Scheme 2). 

The structures of compounds **8** and **10 **were confirmed by the presence of the (two) C=O bands in the IR spectrum at 1,735 and 1,969 cm^−1^ for the acetoxy and amide groups. Compounds **9, 10, 12** and **13** showed characteristic OH group bands at 3,450–3,486 cm^−1^. In addition, the ^1^H-NMR spectrum of **8** showed signals at δ 1.91 and 4.06 ppm characteristic for CH_3_CO and SCH_2_ groups, respectively, while compound **10** showed the deshielded signal at δ 4.45 ppm for the SCH_2_O group. 

**Scheme 3:**
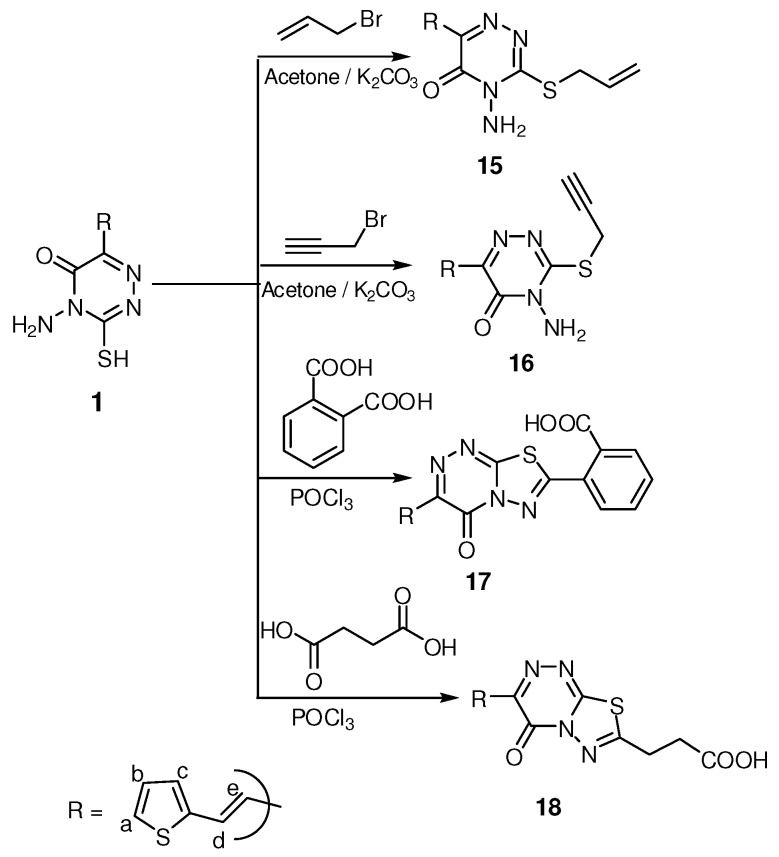
Synthesis of *S* alkyl 1,2,4-triazinone and fused systems.

The ^1^H-NMR spectrum of compound **12** gave signals at δ 1.98, 3.53 and 4.48 ppm characteristic for CH_2(g)_, CH_2(f)_ and CH_2(h)_ groups, in addition to a triplet signal at δ 4.85 ppm for the OH group which exchanged with D_2_O. Its ^13^C-NMR showed signals at δ 30.30, 50.08, 58.02 ppm characteristic for CH_2(g)_, CH_2(f)_ and CH_2(h)_, respectively., while the ^1^H-NMR spectrum of **14** showed signals at δ 4.05–4.18, 4.25 ppm for the CH_2_-O and CH-O of an epoxypropyl moiety, in addition to signals at δ 4.67 and 4.87 ppm for the diastereotropic protons of the S-CH_2 _grouping. IR, ^1^H-NMR and elemental analysis data for compounds **9, 11** and **13** were in agreement with the assigned structures as shown in the Experimental section. Alkylation of compound **1** with allyl and propargyl bromide in the presence of K_2_CO_3_/acetone afforded *S*-alkyl triazine derivatives **15** and **16** (Scheme 3).

The ^1^H-NMR spectrum of **15** showed doublet signals at δ 5.06, 5.24 and 5.31 ppm characteristic for SCH_2_ and the terminal olefin =CH_2_ protons, while its ^13^C-NMR spectrum gave a signal at δ 60.1 ppm for SCH_2_ carbon. Menawhile, the ^13^C-NMR for compound **16** showed signals at δ 48.3 and 76.6, 77.1 ppm for SCH_2_ and C≡C carbons.

In the reaction of compound **1** with phthalic and succinic acids in the presence of POCl_3_, an intramolecular cyclization took place giving the thiadiazolo[2,3-c][1,2,4]triazine derivatives **17** and **18**, respectively (Scheme 3). The structures of compound **17** and **18** were supported by elemental analysis and spectral data. The IR spectra of **17** and **18** showed the absence of the (NH_2_) bands and the presence of C=O and OH bands of acids in the 1,710–1,715 and 3,445–3,463 cm^−1^ range. In addition, the ^1^H-NMR spectra of these compounds revealed the presence of signals at δ 12.18 and 12.28 ppm for the OH group of an acid, while in compound **18** there are two triplet signals at δ 1.82 and 2.68 ppm for the CH_2_-CH_2_ protons, respectively. In addition the ^13^C-NMR spectrum showed signals at δ 26.80 and 32.50 ppm for CH_2_-CH_2_ carbon and eleven sp^2^ signals for the aromatic carbons, three C=N and two C=O groups.

### 2.1. Pharmacological Studies

#### 2.1.1. Cytotoxicity of the compounds against Hep-G2 Cells

**Figure 1:**
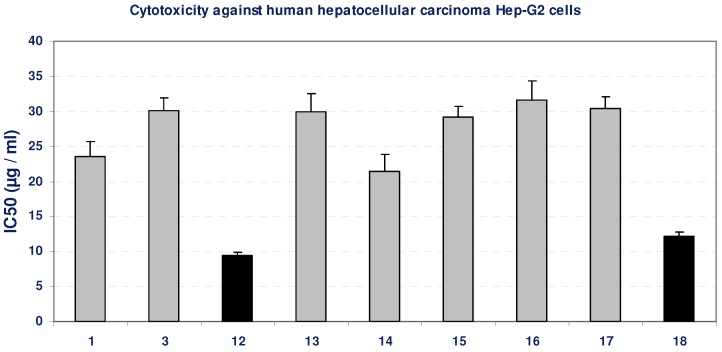
Cytotoxicity (IC_50_, µg/mL) of different tested compounds against human Hep-G2 cells 48 hours of incubation. The grey bars represent non-cytotoxic compounds, and the black bars represent the promising cytotoxic compounds. Data represent mean value of IC_50_ ± SE.

Using the MTT assay we studied the effect of the compounds on the viability of cells after 48 h incubation. Incubation of Hep-G2 cell line with gradual doses of all the compounds led to insignificant change in the growth of Hep-G2 cells, as indicated from their IC_50_ values (>20 µg/mL), except for the compounds **12** and **18**, which produced an inhibition in the viability of Hep-G2 cells compared with the growth of untreated control cells, as concluded from their low IC_50_ values, indicated by black bars in Figure 1. The positive control, paclitaxol, which is a known anti-cancer drug, show high cytotoxicity against Hep-G2 cells with an IC_50_ value of 643 ng/mL (Table 1, Figure 1).

**Table 1:** Cytotoxicity test using MTT assay against three different human cancer cell lines.

Compd. No.	Mean IC_50_ (µg/mL)			SE		
	Hep-G2 cells	MCF-7 cells	HCT-116 cells	Hep-G2 cells	MCF-7 cells	HCT-116 cells
1	23.51	21.04	32.6	2.25	1.62	1.45
3	30.12	39.2	27.23	1.88	2.08	2.70
12	*9.44*	*10.06*	*6.51*	0.45	0.65	0.69
13	30.02	26.78	36.12	2.49	2.07	1.85
14	21.51	25.69	33.67	2.32	1.48	1.77
15	29.14	21.67	18.38	1.54	2.01	1.50
16	31.64	28.88	40.08	2.77	2.18	1.99
17	30.47	21.09	23.86	1.65	2.10	1.46
18	*12.13*	*11.65*	*8.34*	0.58	0.84	0.80

#### 2.1.2. Cytotoxicity of the compounds against MCF-7 Cells

**Figure 2:**
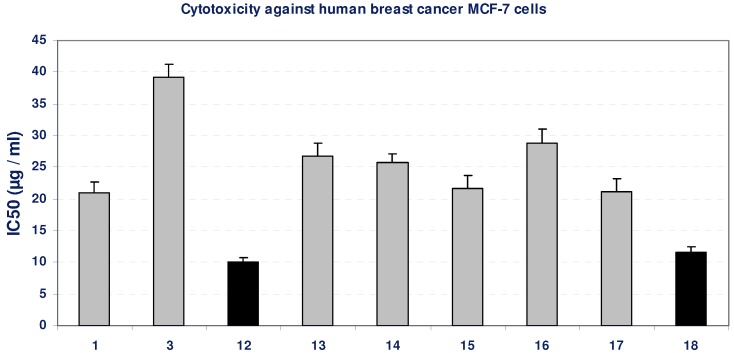
Cytotoxicity (IC50, µg/mL) of different tested compounds against human MCF-7 cells 48 hours of incubation. The grey bars represent non-cytotoxic compounds, and the black bars represent the promising cytotoxic compounds. Data represent mean value of IC_50_ ± SE.

Using the MTT assay we studied the effect of the compounds on the viability of cells after 48 h incubation. Incubation of MCF-7 cell line with most of the tested compounds led to insignificant changes in the growth of MCF-7 cells, as indicated from their IC_50_ values (>20 µg/mL), except for compounds **12** and **18**, which possessed an inhibitory effect on MCF-7 cells viability, compared with the growth of untreated control cells, as concluded from their low IC_50_ values, indicated by black bars in Figure 2. The positive control, paclitaxol, which is a known anti-cancer drug, resulted in high cytotoxicity against MCF-7 cells with IC_50_ value of 452 ng/mL (Table 1, Figure 2).

#### 2.1.3. Cytotoxicity of the compounds against HCT-116 Cells

**Figure 3:**
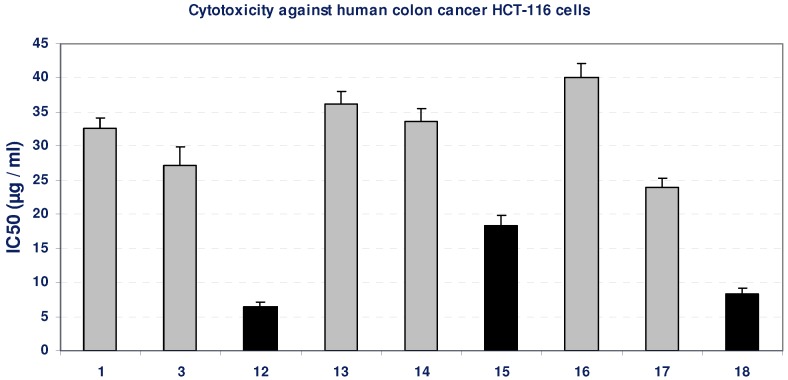
Cytotoxicity (IC50, µg/mL) of different tested compounds against human HCT-116 cells after 48 h of incubation. The grey bars represent non-cytotoxic compounds, and the black bars represent the promising cytotoxic compounds. Data represent mean value of IC_50_ ± SE.

The effect of the compounds on the viability of cells after 48 h incubation was studied by MTT assay. Incubation of HCT-116 cell line with gradual doses of some tested compounds led to insignificant change in the growth of HCT-116 cells as indicated from their IC_50_ values (>20 µg/mL). On the other hand, the compounds **12**, **15** and **18** resulted in a significant inhibition in the viability of HCT-116 cells, compared with the growth of untreated control cells, as concluded from their low IC_50_ values, as indicated by black bars in Figure 3. The positive control, paclitaxol, which is a known anti-cancer drug, resulted in high cytotoxicity against HCT-116 cells with IC_50_ value of 709 ng/mL (Table 1, Figure 3).

#### 2.1.4. Percentage of induced apoptotic and necrotic cells in Hep-G2 Cells

**Figure 4:**
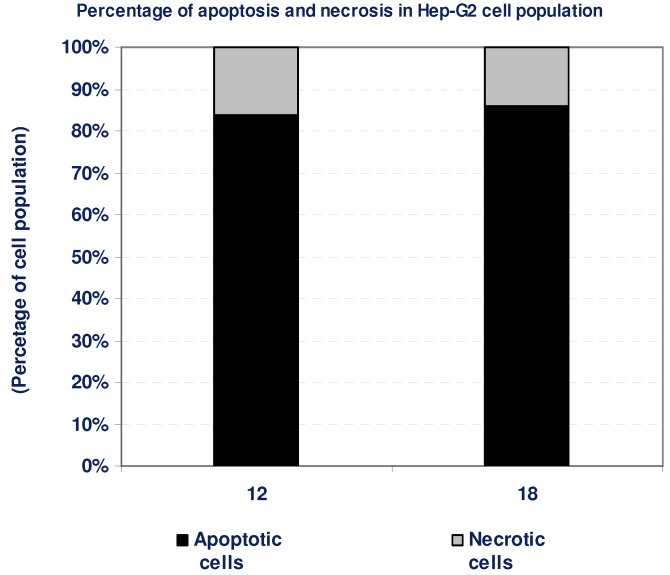
The type of cell death was investigated in Hep-G2 cells after the treatment with the promising cytotoxic compounds, using acridine orange/ethidium bromide staining to compare between the percentage of necrotic cells (grey segment) and the apoptotic cells (black segment). Data represent mean value ± SE.

According to the cytotoxicity experiments, compounds **12** and **18** possessed potent cytotoxic effects against Hep-G2 cells. To detect the type of cell death induced in the cells by those compounds, Hep-G2 cells were treated with the IC_50_ values of each compound for 6 h and the apoptosis and necrosis cell population percentages was recorded using acridine orange/ethidium bromide staining. As shown in Figure 4, both of the tested compounds led to an apoptosis-dependant cell death (84–86% of the total dead cell number), while the percentage of necrotic cells were only 14–16% of the total dead cell number (Table 2, Figure 4).

**Table 2:** Apoptosis and necrosis assay for cytotoxic compounds only.

Compd. No.	Hep-G2	
	Apoptotic cells	Necrotic cells
12	84	16
18	86	14

#### 2.1.5. Percentage of induced apoptotic and necrotic cells in MCF-7 Cells

**Figure 5:**
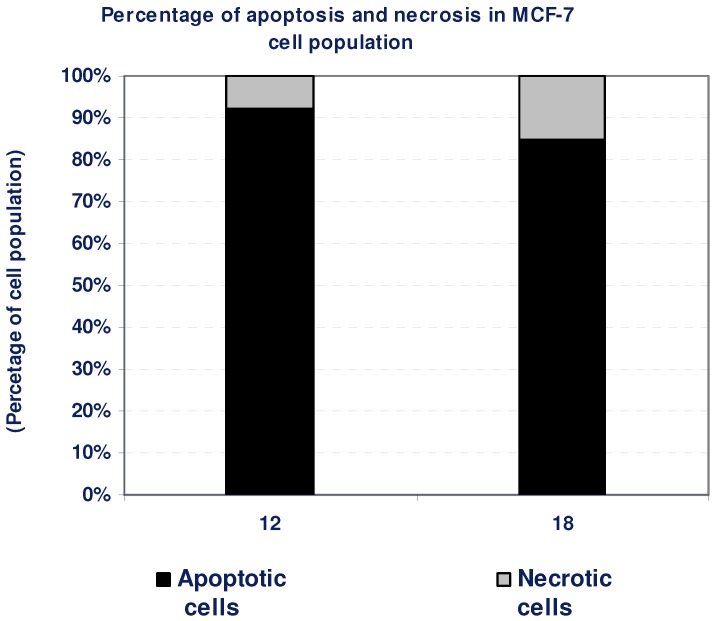
The type of cell death was investigated in MCF-7 cells after the treatment with the promising cytotoxic compounds, using acridine orange/ethidium bromide staining to compare between the percentage of necrotic cells (grey segment) and the apoptotic cells (black segment). Data are representing mean value ± SE.

According to the findings of the cytotoxicity experiment, compounds **12** and **18** possessed a potent cytotoxic effect against MCF-7 cells. To detect the type of cell death induced in the cells by those compounds, MCF-7 cells were treated with the IC_50_ values of each compound for 6 h and the apoptosis and necrosis cell population percentages was recorded using acridine orange/ethidium bromide staining. As shown in Figure 5, both of the tested compounds led to an apoptosis-dependant cell death (85–92% of the total dead cell number), while the percentage of necrotic cells were only 8–15% of the total dead cell number (Table 3, Figure 5).

**Table 3:** Apoptosis and necrosis assay for cytotoxic compounds only.

Compd. No.	MCF-7	
	Apoptotic cells	Necrotic cells
12	92	8
18	85	15

#### 2.1.6. Percentage of induced apoptotic and necrotic cells in HCT-116 Cells

**Figure 6:**
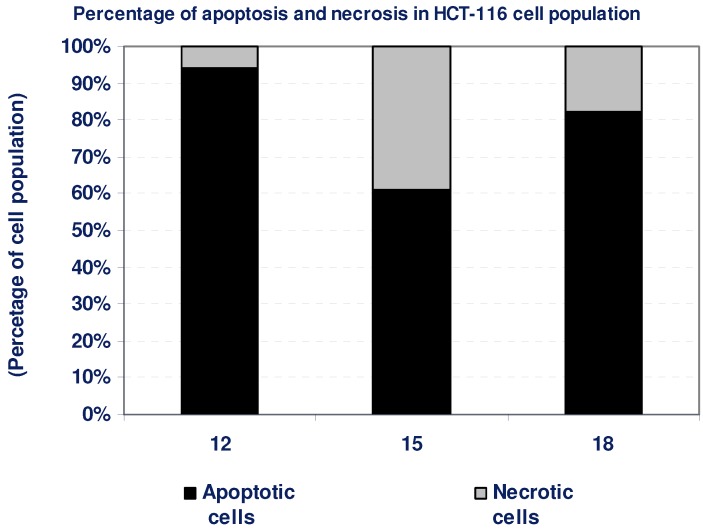
The type of cell death was investigated in HCT-116 cells after the treatment with the promising cytotoxic compounds, using acridine orange/ethidium bromide staining to compare between the percentage of necrotic cells (grey segment) and the apoptotic cells (black segment). Data are representing mean value ± SE.

According to the cytotoxicity experiments, compounds **12**, **15**, and **18** possessed potent cytotoxic effects against HCT-116 cells. To detect the type of cell death induced in the cells by those compounds, HCT-116 cells were treated with the IC_50_ values of each compound for 6 h and the apoptosis and necrosis cell population percentages was recorded using acridine orange/ethidium bromide staining. As shown in Figure 6, the tested compounds **12**, **15**, and **18** resulted in an apoptosis-dependant cell death (61–94% of the total dead cell number), while the percentage of necrotic cells were only 6–39% of the total dead cell number (Table 4, Figure 6).

**Table 4:** Apoptosis and necrosis assay for cytotoxic compounds only.

Compd. No.	HCT-116	
	Apoptotic cells	Necrotic cells
12	94	6
15	61	39
18	82	18

## 3. Experimental

### 3.1. General

All melting points were taken on an Electrothermal IA 9100 series digital melting point apparatus. The IR spectra (KBr) discs were recorded on a Perkin-Elmer 1650 spectrometer. ^1^H-, ^13^C-NMR spectra were determined on Bruker AC-300 MHz instrument. Chemical shifts are expressed as δ (ppm) relative to TMS as internal standard and DMSO-d_6_ as solvent. The elemental analysis and mass spectra were carried by the Micro-analytical Center, Cairo University. Mass spectra were recorded on a Shimadzu GC-MS-QP 1000 EX spectrometer. A domestic microwave oven was used (2,450 MHz, 800 W). The pharmacological studies were carried out in National Research Center (Center of Excellence for Advanced Sciences, Cancer Biology Research Laboratory). All chemicals were from Sigma.

### 3.2. Material and Methods for Pharmacological Activity

#### 3.2.1. Cell culture

Several human cell lines were used in testing the anti-cancer activity including: hepatocellular carcinoma (Hep-G2), colon carcinoma (HCT-116), and histiocytic lymphoma and breast adenocarcinoma (MCF-7) (ATCC, VA, USA). HCT-116 cells were grown in Mc Coy's medium, while all cells were routinely cultured in DMEM (Dulbeco’s Modified Eagle’s Medium) at 37 °C in humidified air containing 5% CO_2_. Media were supplemented with 10% fetal bovine serum (FBS), 2 mM L-glutamine, containing 100 units/mL penicillin G sodium, 100 units/mL streptomycin sulphate, and 250 ng/mL amphotericin B. Monolayer cells were harvested by trypsin/EDTA treatment, while and leukemia cells were harvested by centrifugation. Compound dilutions were tested before assays for endotoxin using Pyrogent® Ultra gel clot assay, and they were found endotoxin free. All experiments were repeated four times, unless mentioned, and the data was represented as (mean ± S.D.). Cell culture material was obtained from Cambrex BioScience (Copenhagen, Denmark), and all chemicals were from Sigma (USA). 

#### 3.2.2. Cytotoxicity assay

Cytotoxicity of tested samples against different types of cells was measured using the MTT Cell Viability Assay. MTT (3-[4,5-dimethylthiazole-2-yl]-2,5-diphenyltetrazolium bromide) assay is based on the ability of active mitochondrial dehydrogenase enzyme of living cells to cleave the tetrazolium rings of the yellow MTT and form a dark blue insoluble formazan crystals which is largely impermeable to cell membranes, resulting in its accumulation within healthy cells. Solubilization of the cells results in the liberation of crystals, which are then solubilized. The number of viable cells is directly proportional to the level of soluble formazan dark blue color. The extent of the reduction of MTT was quantified by measuring the absorbance at 570 nm [38]. 

##### 3.2.2.1. Reagents preparation

MTT solution: 5 mg/mL of MTT in 0.9% NaCl; Acidified isopropanol: 0.04 N HCl in absolute isopropanol.

##### 3.2.2.2. Procedure

Cells (0.5 × 10^5^ cells/ well) in serum-free media were plated in a flat bottom 96-well microplate, and treated with 20 µL of different concentrations of each tested compound for 48 h at 37 °C, in a humidified 5% CO_2_ atmosphere. After incubation, media were removed and 40 µL MTT solution/well were added and Incubated for an additional 4 h. MTT crystals were solubilized by adding 180 µL of acidified isopropanol/well and plate was shacked at room temperature. Followed by photometric determination of the absorbance at 570 nm using microplate ELISA reader. Triplicate repeats were performed for each concentration and the average was calculated. Data were expressed as the percentage of relative viability compared with the untreated cells compared with the vehicle control, with cytotoxicity indicated by <100% relative viability.

##### 3.2.2.3. Calculations

Percentage of relative viability was calculated using the following equation:

[Absorbance of treated cells / Absorbance of control cells)] × 100

Then the half maximal inhibitory concentration IC_50_ was calculated from the equation of the dose response curve.

#### 3.2.3. Apoptosis and necrosis staining

The type of cell death was investigated in compound-treated and untreated cells using acridine orange/ethidium bromide staining [39,40]. In brief, cells were treated with The IC_50_ value of each promising compound for 6 h and collected to be treated with acridine orange/ethedium bromide mixture. The vital, necrotic, and apoptotic cells were counted. A mixture of 100 µg/mL acridine orange and 100 µg/mL ethidium bromide was prepared in PBS. The cell uptake of the stain was monitored under a fluorescence microscope, and the apoptotic, necrotic, and viable cells were counted. The early apoptotic cells had yellow chromatin in nuclei that were highly condensed or fragmented. Apoptotic cells also exhibited membrane blebbing. The late apoptotic cells had orange chromatin with nuclei that were highly condensed and fragmented. The necrotic cells had bright orange chromatin in round nuclei. Only cells with yellow, condensed, or fragmented nuclei were counted as apoptotic cells in a blinded, nonbiased manner.

*4-Amino-3-mercapto-6-[2-(2-thienyl)vinyl]-1,2,4-triazin-5(4H)-one* (**1**). *Method A:* A mixture of 2-oxo-4-(2-thienyl)but-3-enoic acid (0.01 mol), thiocarbohydrazide (0.01 mol) in glacial acetic acid (25 mL) was stirred under reflux for 2 h, cooled to room temperature, the precipitate separated collected by filtration to give a yellowish crystals. Yield 62%; m.p. 252–255 °C. *Method B:* A mixture of 2-oxo-4-(2-thienyl)but-3-enoic acid (0.01 mol) and thiocarbohydrazide (0.01 mol), was dissolved in a mixture of methylene chloride/methanol (80:20, 15 mL) then silica gel (1.0 g, 200–400 mesh) was added, the solvent was removed by evaporation, the dried residue was transferred into a glass beaker and irradiated for (1.5–2.0 min) in a domestic microwave oven (2,450 MHz, 800 W). The product was chromatographed on a silica gel column, using methylene chloride as eluent. Yield 98%; m.p. 254–255 °C; IR (KBr): 3,295–3,201 cm^−1^ (NH_2_), 1,666 cm^−1^ (C=O amide); ^1^H-NMR (DMSO-d_6_): δ = 6.51 (s, 2H, NH_2_), 6.74 (d, 1H, *J* = 15.9 Hz, CH=CH_e_), 7.07 (dd, 1H, *J* = 3.60, 4.80 Hz, thiophene-H_b_) 7.36 (d, 1H, *J* = 3.6 Hz, thiophene–H_c_), 7.57 (d, 1H, *J* = 5.1 Hz, thiophene–H_a_), 7.92 (d, 1H, *J* = 16.0 Hz, CH_d_=CH), 14.00 (s, 1H, SH); ^13^C-NMR (DMSO-d_6_): δ = 118.2, 127.7, 128.3, 128.6, 129.5, 141.0, 141.1, 148.0 and 167.2 (Ar-C, C=C, C=N and C=O); Anal. Calcd for C_9_H_8_N_4_OS_2 _(252.31): C, 42.84; H, 3.20; N, 22.20; found C, 42.90; H, 3.16; N, 22.03; MS *m/e* (int. %): 250 (82.8), 251 (90.8), 250 (100), 135 (87.4), 134 (65.5), 69 (80.5), 59 (82.8), 58 (93.1), 51 (75.9), 50 (62.1).

#### 3.2.4. General method for preparation of compounds **3** and **6**

A mixture of compound **1** (10 mmol) and peracetylated sugar (10 mmol) was dissolved in methylene chloride (20 mL), then silica gel (1 g, 200–400 mesh) was added, the solvent was removed by evaporation and the dried residue was irradiated for 5 min in a domestic microwave oven (2,450 MHz, 800 W). The product was extracted with methylene chloride, evaporated to dryness and purified by recrystallization from ethanol. 

*Method A: 4-Amino-3-(2`,3`,4`,6`-tetra-O-acetyl-β-D-glucopyranosylthio)-6-[2-(2-thienyl)vinyl]-1,2,4-triazin-5(4H)-one* (**3**). Yield 21%; m.p. 86–87 °C; IR (KBr): 3,310 cm^−1^ (NH_2_), 1,739 cm^−1^ (C=O, acetoxy groups), 1,660 cm^−1^ (C=O, amide); ^1^H-NMR (DMSO-d_6_): δ = 1.87, 1.92, 1.95 and 2.02 (4s, 12H, 4 CH_3_CO), 4.03 (m, 2H, H-5` and H-6''), 4.28 (m,1H, H-6'), 5.06 (t, 1H, *J* = 9.80 Hz, H-4'), 5.61 (t, 1H, *J* = 9.80 Hz, H-2'), 5.69 (t, 1H, *J* = 8.65 Hz, H-3'), 6.21 (d, 1H, *J*_1`,2`_ = 9.60 Hz, H-1'), 6.64 (s, 2H, NH_2_, exchanged with D_2_O), 6.76 (d, 1H, *J* = 15.9 Hz, CH=CH_e_), 7.13 (dd, 1H, *J* = 3.90, 4.80 Hz, thiophene-H_b_), 7.45 (d, 1H, *J* = 3.30 Hz, thiophene-H_c_), 7.66 (d, 1H, *J* = 4.80 Hz, thiophene-H_a_), 8.01 (d, 1H, *J* = 15.9 Hz, CH_d_=CH);^ 13^C-NMR (DMSO-d_6_): δ = 19.70, 20.30, 20.46 and 21.14 (4 CH_3_CO), 61.36 (C-6'), 66.24 (C-4'), 68.47 (C-3'), 72.88 (C-2'), 73.13 (C-5') and 86.73 (C-1'), 116.9, 128.4, 130.0, 130.1, 140.3, 140.7, 146.7, 146.8, 168.7, 169.0, 169.4, 169.5 and 170.0 (Ar-C, C=C, 2C=N and 5 C=O); Anal. Calcd for C_23_H_26_N_4_O_10_S_2_ (582.60): C, 47.42; H, 4.50; N, 9.62. Found: C, 47.40; H, 4.50; N, 9.61. *Method B: *A solution of 2,3,4,6-tetra-*O*-acetyl-*β-*D-glucopyranosyl bromide **2** (10 mmol) in acetone (30 mL) was added to a solution of compound **1** (10 mmol) in aqueous (distilled water, 10 mL) potassium hydroxide (10 mmol). The reaction mixture was stirred overnight at room temperature. The solvent was removed under reduced pressure, the residue was washed with distilled water to remove the inorganic residue and then the formed solid product dried and crystallized from ethanol. Yield 25%; m.p. 85–87 °C. 

#### 3.2.5. General procedure for preparation of compounds **4**,**7**,**9** and **10**

A mixture of any of the compounds **3**, **6**, **8** or **10** (10 mmol) in methanol (20 mL), triethylamine (1 mL) and few drops of water were stirred overnight at room temperature, and then the solvent was evaporated under reduced pressure. The residue was purified by crystallization from ethanol.

*4-Amino-3-(β-D-glucopyranosylthio)-6-[2-(2-thienyl)vinyl]-1,2,4-triazin-5(4H)-one* (**4**). Yield 85%; m.p. 140–142 °C; IR (KBr): 3,434 cm^−1^ (OH), 3,225 cm^−1^ (NH_2_), 1,665 cm^−1^ (C=O, amide); ^1^H NMR (DMSO-d_6_): δ = 3.14–3.64 (m, 6H, H-6', H-6'', H-5', H-4', H-3' and H-2'), 4.48 (d, 1H, OH-2'), 5.08 (d, 1H, OH-3'), 5.23 (d, 1H, OH-4'), 5.48 (t, 1H, OH-6'), 5.59 (d, 1H, *J*_1`,2`_ = 7.82 Hz, H-1'), 6.65 (s, 2H, NH_2_), 6.77 (d, 1H, *J* = 15.9 Hz, CH=CH_e_), 7.10 (dd, 1H, *J* = 3.90, 4.80 Hz, thiophene-H_b_), 7.35 (d, 1H, *J* = 3.30 Hz, thiophene-H_c_), 7.65 (d, 1H, *J* = 4.80 Hz, thiophene-H_a_), 8.01 (d, 1H, *J* = 15.9 Hz, CH_d_=CH); Anal. Calcd for C_15_H_18_N_4_O_6_S_2_ (414.46): C, 43.47; H, 4.38; N, 13.52. Found: C, 43.45; H, 4.39; N, 13.50.

*4-Amino-3-(2`,3`,5`-tri-O-acetyl-β-D-ribofuranosylthio)-6-[2-(2-thienyl)vinyl]-1,2,4-triazin-5(4H)-one *(**6**). Yield 20%; m.p. 92–94 °C; IR (KBr): 3,328 cm^−1^ (NH_2_), 1,737 cm^−1^ (C=O, acetoxy groups), 1,675 cm^−1^ (C=O, amide); ^1^H-NMR (DMSO-d_6_): δ = 1.70, 2.11 and 2.14 (3s, 9H, 3 CH_3_CO), 4.09 (m, 2H, H-5' and H-5''), 4.37 (m, 1H, H-4'), 5.44 (t, 1H, *J*_2`,3`_ = 5.90, *J*_3`,4`_ = 6.00 Hz, H-3'), 5.79 (t, 1H, *J* = 2.60 Hz, H-2'), 6.46 (d, 1H, *J*_1`,2`_ = 2.80 Hz, H-1'), 6.52 (s, 2H, NH_2_, exchange with D_2_O), 6.82 (d, 1H, *J* = 15.9 Hz, CH=CH_e_), 7.10 (dd, 1H, *J* = 3.80, 4.82 Hz, thiophene-H_b_), 7.41 (d, 1H, *J* = 3.30 Hz, thiophene-H_c_), 7.60 (d, 1H, *J* = 4.80 Hz, thiophene-H_a_), 8.01 (d, 1H, *J* = 15.9 Hz, CH_d_=CH); ^13^C-NMR (DMSO-d_6_): δ = 20.20, 20.41 and 20.74 (3 CH_3_CO), 63.10 (C-5'), 70.02 (C-3'), 73.54 (C-2'), 79.01 (C-4'), 87.60 (C-1'), 118.3, 127.8, 128.4, 128.6, 129.6, 141.1, 141.2, 148.1, 167.3, 168.7, 169.4 and 169.9 (Ar-C, C=C, 2 C=N and 4 C=O); Anal. Calcd for C_20_H_22_N_4_O_8_S_2_ (510.54): C, 47.05; H, 4.34; N, 10.97. Found: C, 47.04; H, 4.35; N, 10.98.

*4-Amino-3-(β-D-ribofuranosylthio)-6-(2-(2-thienyl)vinyl)-1,2,4-triazin-5(4H)-one* (**7**). Yield 85%; m.p. 137–139 °C; IR (KBr): 3,421 cm^−1^ (OH), 3,280 cm^−1^ (NH_2_), 1,668 cm^−1^ (C=O, amide);^ 1^H-NMR (DMSO-d_6_): δ = 3.98–4.07 (m. 5H, H-5', H-5'', H-4', H-3' and H-2'), 4.20 (t, 1H, OH-5'), 4.26 (d, 1H, OH-3'), 4.30 (d, 1H, OH-2'), 6.03 (d, 1H, *J*_1`,2`_ = 3.80 Hz, H-1'), 6.64 (s, 2H, NH_2_), 6.75 (d, 1H, *J* = 15.9 Hz, CH=CH_e_), 7.09 (dd, 1H, *J *= 3.80, 4.82 Hz, thiophene-H_b_), 7.34 (d, 1H, *J* = 3.30 Hz, thiophene-H_c_), 7.63 (d, 1H, *J* = 4.80 Hz, thiophene-H_a_), 8.10 (d, 1H, *J* = 15.9 Hz, CH_d_=CH); Anal. Calcd for C_14_H_16_N_4_O_5_S_2_ (384.43): C, 43.74; H, 4.20; N, 14.57. Found: C, 43.76; H, 4.22; N, 14.56.

#### 3.2.6. General procedure for synthesis of S-alkyl compounds **8**, **10**, **12**, **13** and **14**

*Method A: *An appropriate alkyl halide such as 4-bromobutylacetate, 2-acetoxyethoxymethyl bromide, 3-chloropropanol, 1,3-dichloro-2-propanol and/or epichlorohydrin (10 mmol) was added to a well stirred mixture of 1 (10 mmol) and potassium carbonate (10 mmol) in dry DMF (15 mL). The reaction mixture was stirred overnight at room temperature, the mixture was filtered off, and the solvent evaporated under reduced pressure. The residue was crystallized from ethanol. *Method B:* The appropriate alkyl halide (10 mmol) was added to a well stirred mixture of **1** (10 mmol) and potassium carbonate (10 mmol) in dry DMF (15 mL). The reaction mixture was stirred under reflux for 2–8 h, the mixture was filtered off, and the solvent evaporated under reduced pressure. The residue was crystallized from ethanol.

*4-(4-Amino-5-oxo-6-(2-(2-thienyl)vinyl)-4,5-dihydro-1,2,4-triazin-3-ylthio)butyl acetate* (**8**). Method B: Yield 60%; m.p. 90–92 °C; IR (KBr): 3280 cm^−1^ (NH_2_), 1735 cm^−1^ (C=O, acetoxy), 1668 cm^−1^ (C=O, amide);^ 1^H-NMR (DMSO-d_6_): δ = 1.63 (m, 2H, CH_2_), 1.83 (m, 2H, CH_2_), 1.91 (s, 3H, CH_3_CO), 4.06 (t, 2H, *J* = 6.60 Hz, SCH_2_), 4.49 (t, 2H, *J* = 6.90 Hz, CH_2_OCO), 6.67 (s, 2H, NH_2_, exchanged with D_2_O), 6.76 (d, 1H, *J* = 15.9 Hz, CH=CH_e_), 7.10 (dd, 1H, *J* = 3.80, 4.80 Hz, thiophene-H_b_), 7.35 (d, 1H, *J* = 3.30 Hz, thiophene-H_c_), 7.61 (d, 1H, *J* = 4.80 Hz, thiophene-H_a_), 8.06 (d, 1H, *J* = 15.9 Hz, CH_d_=CH); Anal. Calcd for C_15_H_18_N_4_O_3_S_2_ (366.46): C, 49.16; H, 4.95; N, 15.29. Found: C, 49.18; H, 4.96; N, 15.28.

*4-Amino-3-(4-hydroxybutylthio)-6-(2-(2-thienyl)vinyl)-1,2,4-triazin-5(4H)-one *(**9**). Yield 85%; m.p. 125–126 °C; IR (KBr): 3,450 cm^−1^ (OH), 3,228 cm^−1^ (NH_2_), 1,665 cm^−1^ (C=O, amide);^ 1^H- NMR (DMSO-d_6_): δ = 1.58 (m, 2H, CH_2_), 1.75 (m, 2H, CH_2_), 3.96 (t, 2H, *J* = 6.62 Hz, SCH_2_), 4.38 (t, 2H, *J* = 6.82 Hz, CH_2_O), 4.82 (br, 1H, OH), 6.68 (s, 2H, NH_2_), 6.75 (d, 1H, *J* = 15.9 Hz, CH=CH_e_), 7.12 (dd, 1H, *J* = 3.80, 4.80 Hz, thiophene-H_b_), 7.41 (d, 1H, *J* = 3.30 Hz, thiophene-H_c_), 7.63 (d, 1H, *J* = 4.80 Hz, thiophene-H_a_), 8.08 (d, 1H, *J* = 15.9 Hz, CH_d_=CH); Anal. Calcd for C_13_H_16_N_4_O_2_S_2_ (324.42): C, 48.13; H, 4.97; N, 17.27.Found: C, 48.15; H, 4.96; N, 17.29.

*2-((4-Amino-5-oxo-6-(2-(2-thienyl)vinyl)-4,5-dihydro-1,2,4-triazin-3-ylthio)methoxy)ethyl acetate* (**10)**. Method A: Yield 60%; m.p. 85–86°C; IR (KBr): 3,295 cm^−1^ (NH_2_), 1,735 cm^−1^ (C=O, acetoxy), 1,669 cm^−1^ (C=O, amide); ^1^H-NMR (DMSO-d_6_): δ = 1.94 (s, 3H, CH_3_CO), 3.72 (t, 2H, *J* = 5.80 Hz, OCH_2_), 4.28 (t, 2H, *J* = 6.00 Hz, CH_2_OCO), 4.45 (s, 2H, SCH_2_O), 6.65 (s, 2H, NH_2_, exchanged with D_2_O), 6.72 (d, 1H, *J* = 15.9 Hz, CH=CH_e_), 7.09 (dd, 1H, *J* = 3.80, 4.80 Hz, thiophene-H_b_), 7.39 (d, 1H, *J* = 3.30 Hz, thiophene-H_c_), 7.58 (d, 1H, *J* = 4.80 Hz, thiophene-H_a_), 8.03 (d, 1H, *J* = 15.9 Hz, CH_d_=CH);^ 13^C-NMR (DMSO-d_6_): δ = 20.5 (CH_3_CO), 61.7 (OCH_2_), 62.9 (CH_2_OCO), 63.04 (SCH_2_O), 117.6, 128.1, 128.4, 129.9, 139.5, 140.8, 146.8, 147.1, 168.9 and 170.2 (Ar-C, C=C, 2 C=N and 2 C=O); Anal. Calcd for C_14_H_16_N_4_O_4_S_2_ (368.43): C, 45.64; H, 4.38; N, 15.21. Found: C, 45.65; H, 4.36; N, 15.20.

*4-Amino-3-((2-hydroxyethoxy)methylthio)-6-(2-(2-thienyl)vinyl)-1,2,4-triazin-5(4H)-one* (**11**). Yield 90%; m.p. 128–130 °C; IR (KBr): 3,486 cm^−1^ (OH), 3,275 cm^−1^ (NH_2_), 1,672 cm^−1^ (C=O, amide);^ 1^H- NMR (DMSO-d_6_): δ = 3.68 (t, 2H, *J* = 5.82 Hz, OCH_2_), 4.08 (t, 2H, *J* = 6.00 Hz, CH_2_OH), 4.50 (s, 2H, SCH_2_O), 4.82 (br, 1H, OH), 6.62 (s, 2H, NH_2_), 6.68 (d, 1H, *J* = 15.9 Hz, CH=CH_e_), 7.10 (dd, 1H, *J* = 3.80, 4.80 Hz, thiophene-H_b_), 7.36 (d, 1H, *J* = 3.30 Hz, thiophene-H_c_), 7.53 (d, 1H, *J* = 4.80 Hz, thiophene-H_a_), 8.10 (d, 1H, *J* = 15.9 Hz, CH_d_=CH); Anal. Calcd for C_12_H_14_N_4_O_3_S_2_ (326.39): C, 44.16; H, 4.32; N, 17.17. Found: C, 44.18; H, 4.34; N, 17.18. 

*4-Amino-3-(3-hydroxypropylthio)-6-(2-(2-thienyl)vinyl)-1,2,4-triazin-5(4H)-*one (**12**). Method B: Yield 50%; m.p. 125–127 °C; IR (KBr): 3,450 cm^−1^ (OH), 3,281 cm^−1^ (NH_2_), 1,683 cm^−1^ (C=O, amide); ^1^H- NMR (DMSO-d_6_): δ = 1.98 (m, 2H, CH_2(g)_), 3.53 (t. 2H, *J* = 6.30 Hz, CH_2(f)_), 4.48 (t, 2H, *J* = 6.90 Hz, CH_2(h)_), 4.85 (t, 1H, *J* = 5.80 Hz, OH), 6.68 (s, 2H, NH_2_), 6.77 (d, 1H, *J* = 15.9 Hz, CH=CH_e_), 7.11 (dd, 1H, *J* = 3.80, 4.80 Hz, thiophene-H_b_), 7.43 (d, 1H, *J* = 3.30 Hz, thiophene-H_c_), 7.62 (d, 1H, *J* = 4.80 Hz, thiophene-H_a_), 8.02 (d, 1H, *J* = 15.9 Hz, CH_d_=CH);^ 13^C-NMR (DMSO-d_6_): δ = 30.30 (CH_2(g)_), 50.08 (CH_2(f)_), 58.02 (CH_2(h)_), 118.1, 128.0, 128.5, 129.4, 129.8, 140.0, 140.9, 147.0 and 166.4 (Ar-C, C=C, 2 C=N and C=O); Anal. Calcd for C_12_H_14_N_4_O_2_S_2_ (310.40): C, 46.43; H, 4.55; N, 18.05. Found: C, 46.44; H, 4.53; N, 18.03.

*4-Amino-3-(3-chloro-2-hydroxypropylthio)-6-(2-(2-thienyl)vinyl)-1,2,4-triazin-5(4H)-one* (**13**). Method B: yield 55%; m.p. 160–162 °C; IR (KBr): 3,452 cm^−1^ (OH), 3,280 cm^−1^ (NH_2_), 1,672 cm^−1^ (C=O, amide); ^1^H-NMR (DMSO-d_6_): δ = 2.90 (m, 1H, H_(g)_), 3.18 (m. 1H, H_(f)_), 3.42 (m, 1H, H_(j)_), 3.68 (m, 1H, H_(i)_), 3.82 (m, 1H, H_(h)_), 4.81 (d, 1H, *J* = 5.30 Hz, OH), 6.62 (s, 2H, NH_2_), 6.71 (d, 1H, *J* = 15.9 Hz, CH=CH_e_), 7.10 (dd, 1H, *J *= 3.80, 4.80 Hz, thiophene-H_b_), 7.35 (d, 1H, *J* = 3.30 Hz, thiophene- H_c_), 7.60 (d, 1H, *J* = 4.80 Hz, thiophene-H_a_), 8.06 (d, 1H, *J* = 15.9 Hz, CH_d_=CH); Anal. Calcd for C_12_H_13_ClN_4_O_2_S_2_ (344.84): C, 41.80; H, 3.80; N, 16.25. Found: C, 41.81; H, 3.82; N, 16.23.

*4-Amino-3-(oxiran-2-ylmethylthio)-6-(2-(2-thienyl)-1,2,4-triazin-5(4H)-one* (**14**). Method A: Yield 65%; m.p. 130–132 °C; IR (KBr): 3,285 cm^−1^ (NH_2_), 1,672 cm^−1^ (C=O, amide); ^1^H-NMR (DMSO-d_6_): δ = 4.05–4.18 (m, 2H, CH_2_O, oxirane ring), 4.25 (m, 1H, CH-O, oxirane ring), 4.67 (t, 1H, *J* = 5.52, 1.80 Hz, SCHH, diastereotropic proton), 4.87 (d, 1H, *J* = 5.33 Hz, SCHH, diastereotropic proton), 6.54 (s, 2H, NH_2_, exchange with D_2_O), 6.67 (d, 1H, *J* = 15.9 Hz, CH=CH_e_), 7.11 (dd, 1H, J = 3.80, 4.80 Hz, thiophene-H_b_), 7.41 (d, 1H, *J* = 3.30 Hz, thiophene-H_c_), 7.62 (d, 1H, *J* = 4.80 Hz, thiophene-H_a_), 8.08 (d, 1H, *J* = 15.9 Hz, CH_d_=CH); Anal. Calcd for C_12_H_12_N_4_O_2_S_2_ (308.38): C, 46.74; H, 3.92; N, 18.17. Found: C, 46.73; H, 3.90; N, 18.18.

#### 3.2.7. General procedure for synthesis of compounds **15** and **16**

The appropriate alkyl halide (10 mmol) allyl bromide and/or propargyl bromide was added portion- wise to a well stirred mixture of **1** (10 mmol) and potassium carbonate (10 mmol) in dry acetone (15 mL). The reaction mixture was stirred overnight at room temperature, the mixture was filtered off, and the solvent evaporated under reduced pressure. The residue was crystallized from ethanol.

*3-(Allylthio)-4-amino-6-(2-(2-thienyl)vinyl)-1,2,4-triazin-5(4H)-one* (**15**). Yield 62%; m.p. 123–125 °C; IR (KBr): 3,280 cm^−1^ (NH_2_), 1,668 cm^−1^ (C=O, amide); ^1^H-NMR (DMSO-d_6_): δ = 5.06 (d, 2H, *J* = 5.10 Hz, SCH_2_), 5.24 (d, 1H, *J* = 3.00 Hz, =CHH), 5.31 (d, 1H, *J* = 10.8 Hz, =CHH), 5.96 (m, 1H, CH=CH_2_), 6.67 (s, 2H, NH_2_, exchanged with D_2_O), 6.76 (d, 1H, *J* = 15.9 Hz, CH=CH_e_), 7.10 (dd, 1H, *J* = 3.90, 4.80 Hz, thiophene-H_b_), 7.43 (d, 1H, *J* = 3.30 Hz, thiophene-H_c_), 7.62 (d, 1H, *J* = 4.80 Hz, thiophene-H_a_), 8.01 (d, 1H, *J* = 15.9 Hz, CH_d_=CH); ^13^C-NMR (DMSO-d_6_): δ = 60.1 (SCH_2_), 118.0, 118.5, 128.1, 128.5, 129.5, 129.8, 131.0, 140.3, 140.9, 147.0 and 166.6 (Ar-C, C=C, 2C=N and C=O); Anal. Calcd for C_12_H_12_N_4_OS_2_ (292.38): C, 49.29; H, 4.14; N, 19.16. Found: C, 49.28; H, 4.15; N, 19.15.

*4-Amino-3-(prop-2-ynylthio)-6-(2-(2-thienyl)vinyl)-1,2,4-triazin-5(4H)-one* (**16**). Yield 70%; m.p. 160–162°C; IR (KBr): 3,295 cm^−1^ (NH_2_), 1,662 cm^−1^ (C=O, amide);^ 1^H-NMR (DMSO-d_6_): δ = 3.45 (t, 1H, *J* = 1.50 Hz, ≡CH), 5.24 (d, 2H, *J* = 2.40 Hz, SCH_2_), 6.65 (s, 2H, NH_2_, exchanged with D_2_O), 6.78 (d, 1H, *J* = 15.9 Hz, CH=CH_e_), 7.01 (dd, 1H, *J* = 3.90, 4.80 Hz, thiophene-H_b_), 7.41 (d, 1H, *J* = 3.30 Hz, thiophene-H_c_), 7.65 (d, 1H, *J* = 4.80 Hz, thiophene-H_a_), 8.08 (d, 1H, *J* = 15.9 Hz, CH_d_=CH); ^13^C-NMR (DMSO-d_6_): δ = 48.3 (SCH_2_), 76.6, 77.1 (C≡C), 117.6, 120.5, 127.5, 128.3, 129.7, 130.1, 141.2, 147.0 and 166.5 (Ar-C, C=C, 2C=N and C=O); Anal. Calcd for C_12_H_10_N_4_OS_2_ (290.36): C, 49.64; H, 3.47; N, 19.30. Found: C, 49.65; H, 3.49; N, 19.30.

#### 3.2.8. General procedure for compounds **17** and **18**

Compound **1** (10 mmol) and the appropriate dicarboxylic acid (10 mmol) in POCl_3_ (15 mL) were gently heated for 6 h, the reaction mixture was concentrated to 1/3 volume under reduced pressure and leave to cool, cold water (20 mL) was added to the reaction mixture at 0 °C, and the solid product that formed was collected by filtration and crystallized from ethanol.

*2-(4-Oxo-3-(2-(2-thienyl)vinyl)-4H-[1,3,4]thiadiazolo-[2,3-c][1,2,4]tri-azin-7-yl)benzoic acid* (**17**). Yield 60%; m.p. 300–302 °C; IR (KBr): 3,463 cm^−1^ (br, OH, acid), 1,715 cm^−1^ (C=O, acid), 1,680 cm^−1^ (C=O, amide); ^1^H-NMR (DMSO-d_6_): δ = 6.72 (d, 1H, *J* = 15.9 Hz, CH=CH_e_), 6.98 (dd, 1H, *J* = 3.80, 4.80 Hz, thiophene-H_b_), 7.38 (d, 1H, *J* = 3.30 Hz, thiophene-H_c_), 7.56 (d, 1H, *J* = 4.80 Hz, thiophene-H_a_), 7.72–8.37 (m, 4H, Ar-H and CH_d_=CH), 12.18 (s, 1H, OH, exchange with D_2_O); Anal. Calcd for C_17_H_10_N_4_O_3_S_2_ (382.42): C, 53.39; H, 2.64; N, 14.65. Found: C, 53.38; H, 2.63; N, 14.64.

*3-(4-Oxo-3-(2-(2-thienyl)vinyl)-4H-[1,3,4]thiadiazolo-[2,3-c][1,2,4]tr-iazin-7-yl)propanoic acid* (**18**). Yield 60%, m.p. 340–342 °C; IR (KBr): 3,445 cm^−1^ (br, OH, acid), 1,715 cm^−1^ (C=O, acid), 1,667 cm^−1^ (C=O, amide); ^1^H-NMR (DMSO-d_6_): δ = 1.82 (t, 2H, CH_2_), 2.68 (t, 2H, CH_2_CO), 6.75 (d, 1H, *J* = 15.9 Hz, CH=CH_e_), 7.09 (dd, 1H, *J* = 3.80, 4.80 Hz, thiophene-H_b_), 7.32 (d, 1H, *J* = 3.30 Hz, thiophene-H_c_), 7.59 (d, 1H, *J* = 4.80 Hz, thiophene-H_a_), 7.99 (d, 1H, *J* = 15.9 Hz, CH_d_=CH), 12.28 (s, 1H, OH, exchanged with D_2_O);^ 13^C-NMR (DMSO-d_6_): δ = 26.80 (CH_2_), 32.50 (CH_2_CO), 118.2, 127.7, 128.4, 128.6, 129.6, 141.0, 141.2, 148.0, 148.5, 165.8 and 167.2 (Ar-C, C=C, 3C=N and 2C=O); Anal. Calcd for C_13_H_10_N_4_O_3_S_2_ (334.37): C, 46.70; H, 3.01; N, 16.76. Found: C, 46.72; H, 3.00; N, 16.77. 

## 4. Conclusions

In this work we prepared the *S*-glycosyl and* S*-alkyl derivatives of 1,2,4-triazinone, in addition to thiadiazolotriazines. From the anticancer tested compounds its revealed that compounds **12** and **18** are active cytotoxic agents against different cancer cell lines, to a variable extent. This cytotoxic effect was found to be mainly due to apoptosis, which indicated that those compounds may act as promising candidate anticancer agents. From the chemistry point of view, the cytotoxic effects may be due to the presence of free CH_2_CH_2_CH_2_OH or CH_2_CH_2_COOH groups in these compounds.
